# Cardiovascular disease risk assessment in patients with rheumatoid arthritis: A scoping review

**DOI:** 10.1007/s10067-024-06996-3

**Published:** 2024-05-11

**Authors:** Louise Murphy, Mohamad M. Saab, Nicola Cornally, Sheena McHugh, Patrick Cotter

**Affiliations:** 1 Catherine McAuley School of Nursing and Midwifery, University College Cork, Cork, Ireland; 2 Department of Rheumatology, Cork University Hospital, Wilton, Cork Ireland; 3 School of Public Health, University College Cork, Cork, Ireland

**Keywords:** Cardiovascular disease, Rheumatoid arthritis, Scoping review, Cardiovascular risk assessment, Patient assessment, Routine care

## Abstract

Patients with rheumatoid arthritis (RA) have an increased risk of developing cardiovascular disease (CVD). Identification of at-risk patients is paramount to initiate preventive care and tailor treatments accordingly. Despite international guidelines recommending all patients with RA undergo CVD risk assessment, rates remain suboptimal. The objectives of this review were to map the strategies used to conduct CVD risk assessments in patients with RA in routine care, determine who delivers CVD risk assessments, and identify what composite measures are used. The Joanna Briggs Institute methodological guidelines were used. A literature search was conducted in electronic and grey literature databases, trial registries, medical clearing houses, and professional rheumatology organisations. Findings were synthesised narratively. A total of 12 studies were included. Strategies reported in this review used various system-based interventions to support delivery of CVD risk assessments in patients with RA, operationalised in different ways, adopting two approaches: (a) multidisciplinary collaboration, and (b) education. Various composite measures were cited in use, with and without adjustment for RA. Results from this review demonstrate that although several strategies to support CVD risk assessments in patients with RA are cited in the literature, there is limited evidence to suggest a standardised model has been applied to routine care. Furthermore, extensive evidence to map how health care professionals conduct CVD risk assessments in practice is lacking. Research needs to be undertaken to establish the extent to which healthcare professionals are CVD risk assessing their patients with RA in routine care.

**Table:** 

**Key Points** • *A limited number of system-based interventions are in use to support the delivery of CVD risk assessments in patients with RA*. • *Multidisciplinary team collaboration, and education are used to operationalise interventions to support Health Care Professionals in conducting CVD risk assessments in practice*. • *The extent to which Health Care Professionals are CVD risk assessing their patients with RA needs to be established*.

**Supplementary Information:**

The online version contains supplementary material available at 10.1007/s10067-024-06996-3.

## Introduction

Rheumatoid Arthritis (RA) is a chronic, destructive, musculoskeletal disorder of unknown aetiology, characterised by symmetric polyarthritis and erosive synovitis [1]. RA age-standardised prevalence rates are rising globally [2], with current rates ranging from 0.24 to 1% of the general population [3]. Despite significant advancements in treatments over the past 20 years, RA still causes substantial mortality due to comorbidities such as cardiovascular disease (CVD) [4]. Studies have demonstrated 45 to 60% increased mortality in RA patient groups due to CVD compared to the general adult population [5–7]. This can be attributed to a high prevalence of traditional risk factors which, when combined with chronic inflammation, result in accelerated atherosclerosis [8, 9]. CVD as an umbrella term represents four separate disease entities: ischemic heart disease, cerebrovascular disease, peripheral arterial disease, and aortic atherosclerosis [10]. Ischemic heart disease and cerebrovascular disease are the top two causes of death globally, with eight out of 10 events being preventable [11].

Due to the increased global prevalence of RA and associated costs to healthcare, particularly in the presence of concomitant CVD, preventative care is vital [12, 13]. In patients with RA, CVD risk screening can be undertaken by any healthcare professional (HCP) involved in patient care including physicians, nurses, and allied health professionals working in a variety of disciplines including but not limited to rheumatology, cardiology, vascular medicine, neurology, and general medicine. CVD risk screening is undertaken to detect the presence or absence of traditional CVD risk factors such as smoking, hyperlipidaemia, or hypertension. CVD risk assessment, however, is the next step in identifying those patients who are deemed ‘at-risk’ of developing a cardiovascular event and stratifies that risk into low, medium, high, or very high, so interventions can be tailored accordingly [14]. CVD risk assessment, therefore, involves the application of an instrument as a composite measure of risk factor variables, and the recording of a score to assess risk beyond the initial screening for traditional risk factors [15]. European guidelines recommend that in patients with RA, a CVD risk assessment using a composite measure of risk factor variables should be performed at least once every five years and following a major change in anti-rheumatic medication [16].

The use of a validated composite measure as part of a standardised approach to CVD risk assessment in patients with RA enables an accurate risk prediction for individual patients [16]. This allows HCPs to inform patients about their prognosis and permits personalised treatment decisions for CVD prevention [17]. Numerous CVD risk assessment measures exist, from general population measures with and without adaptation for RA, to RA disease specific tools, examples of which can be seen in Online Resource 1. EULAR (European Alliance of Associations for Rheumatology, formally the European League Against Rheumatism) published recommendations on CVD risk management in patients with RA [16]. These recommendations suggest all patients with RA should be CVD risk assessed using a CVD risk assessment measure, recommended by either national or international guidelines. Furthermore, EULAR recommends all risk prediction algorithms not including RA as an independent variable should be adjusted by a 1.5 multiplication factor to enable more accurate risk prediction estimates in this patient cohort [16].

The delivery of CVD risk assessments, beyond the use of a composite measure has not been well defined. Supportive strategies may be in use to assist HCPs in delivering CVD risk assessments in practice. Therefore, the aim of this review was to identify the scope of literature available regarding CVD risk assessments undertaken by HCPs as part of routine care in patients with RA.

The objectives of this review were to:

I. Map the strategies HCPs use to deliver CVD risk assessment in patients with RA.
II. Determine how and who conducts a CVD risk assessment in patients with RA.
III. Identify what composite measures are used in practice when assessing patients with RA for CVD risk.

## Methods

This scoping review applied the Joanna Briggs Institute Methodological Guidelines [18]. A protocol for this review was registered with the Open Science Framework (https://osf.io/f68vu). The Preferred Reporting Items for Systematic Reviews and Meta-Analysis extension for Scoping Reviews (PRISMA-ScR) checklist [19] was used to guide the reporting of this review and is available in Online Resource 2.

### Eligibility criteria

The population, concept, and context (PCC) framework was used to determine the review eligibility criteria [18]. These were as follows: Population: Any HCP involved in the care of patients with RA; Concept: CVD risk assessment, including risk assessment using a risk prediction instrument as a composite measure of risk factors, as part of a risk management strategy, or risk prediction study; Context: Patients aged 18 years and older with RA cared for in any setting. We sought to include experimental and epidemiological studies including randomised and non-randomised controlled trials, quasi-experimental studies, prospective and retrospective cohort studies, case reports, cross-sectional studies, qualitative research, and grey literature such as policy statements, and government reports that yield data relating to CVD risk assessment in patients with RA as part of routine care. In this review, routine care was defined as an established way of working at the time a study or report was undertaken [20], as opposed to experimental systems introduced as part of an intervention for the study period only. Routine care encompasses patient centred centre, and is focused on individual patients, conducted by HCPs, in real-world settings, rather than investigative studies that tend to be focused on populations, involving researchers and research subjects, conducted under experimental conditions [21]. We included interventional studies where the intervention delivered CVD risk assessments in a routine care setting or where CVD risk assessment was part of routine care and the intervention aimed to enhance risk assessment rates or improve patient outcomes.

Studies involving patients with other forms of inflammatory joint disease where RA data couldn’t be isolated were excluded. Studies that focused on single risk factor prediction models or individual risk factor correlation/ incidence/ prevalence studies were also excluded. Studies where CVD risk assessment scoring was researcher led and conducted as part of the study intervention, rather than clinician led as part of routine care were excluded. Data relating to participants younger than 18 years of age were also excluded.

### Information sources

Searches for peer-reviewed publications were conducted in six electronic databases (MEDLINE and CINAHL via EBSCO, the Cochrane Database of Systematic Reviews, Scopus, Web of Science and Academic Search Complete). Three trial registries (ClinicalTrials.gov, EU Clinical Trial Register, and the International Clinical Trials Registry Platform) were also searched to capture any relevant information.

Three grey literature databases (Base, OAIster, and Trip Pro) were searched for information relating to standard methods of CVD risk assessment in patients with RA internationally. Databases of relevant major medical clearing houses (Lenus, the National Guidelines Clearing House, and the Guidelines International Network) were also searched for information relating to policy documents or recommendations. Of note, grey literature searching was conducted with a focus on countries that score high on the human development index (HDI) and that possess similar healthcare development rankings such as life expectancy, education, and gross income per capita [22]. These countries include Ireland, the United Kingdom (UK), Denmark, Sweden, the Netherlands, the United States of America (USA), Canada, Australia, and New Zealand [23].

Considering the target population (i.e., patients with RA), further grey literature specific to the discipline of rheumatology was sought from the International League of Associations for Rheumatology website. This helped identify relevant information from regional partner organisations including EULAR, the American College of Rheumatology, the African League of Associations for Rheumatology, the Asia Pacific League of Associations for Rheumatology, and the Pan American League of Associations for Rheumatology.

### Search strategy

Relevant keywords and subject headings were identified for CVD, risk assessment, and RA (Online Resource 3). Searches were last conducted in December 2023. The search strategy for each database is available in Online Resource 4. Searches were customised to all peer-reviewed electronic databases and limited to the English language. No date or setting limitations were applied to maximise retrieval. The reference lists of all sources deemed eligible and included in the review were searched for additional relevant studies.

### Selection of sources of evidence

The web-based software tool Covidence was used to screen and select relevant studies from all information sources [24]. Duplicates were deleted automatically in Covidence. Title, abstract, and full text screenings were conducted independently by two authors (LM and either PC, MMS, or NC) and conflicts were resolved by a third.

### Charting the data

A data extraction tool adapted from the Joanna Briggs Institute [18] was utilised to extract key information about the sources of evidence regarding the author(s), year of publication, country, aims, design, sample size and target population, CVD assessment delivery including any strategy used, who the assessment was conducted by and in what location, composite measure(s) used (including modifications), frequency of assessment, and study findings. Data extraction was completed by LM and checked for accuracy by PC, MMS, and NC.

### Synthesis of results

A narrative synthesis was conducted from the extracted data using a priori determined headings guided by the objectives of this review and included: strategies used to CVD risk assess patients with RA, HCPs who conduct CVD risk assessments in patients with RA, the setting where CVD risk assessments took place, the composite measure used, if adjusted for RA, and frequency of measure application.

## Results

### Selection of sources of evidence

**Fig. 1:**
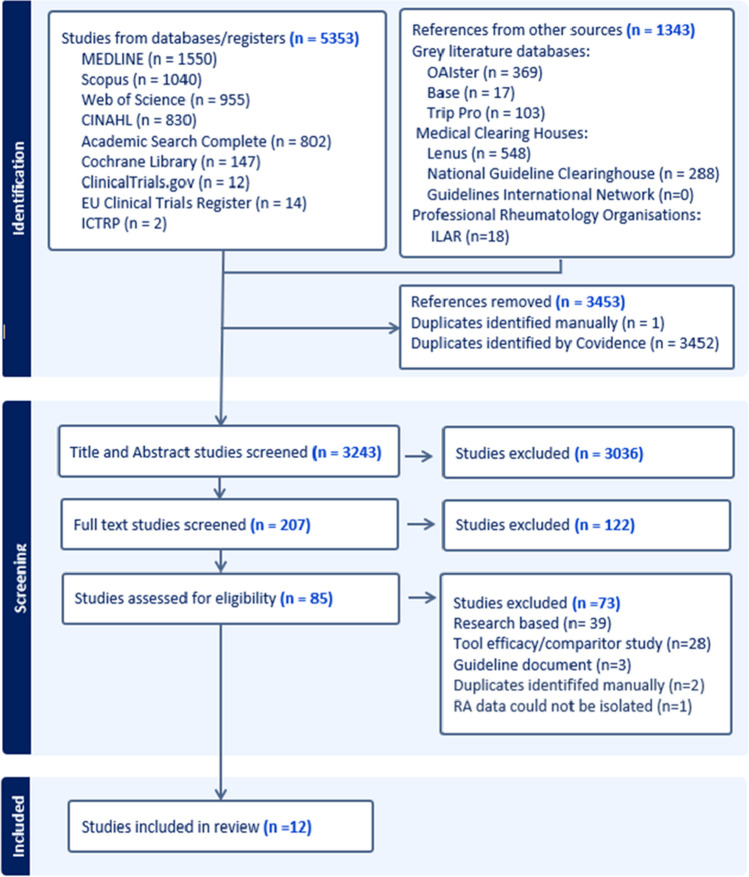
PRISMA flow chart

The initial search following deduplication yielded 3,243 results. After title and abstract screening, 207 records were included for full text review. Of those, 12 records were deemed eligible for inclusion in the review. All records were identified from electronic database searching. No records were included from trial registries or the grey literature search. Further details on study selection can be found in the PRISMA flowchart (Fig. 1).

### Results of individual sources of evidence

Findings from the included studies, including study characteristics are available in the data extraction table (Table 1).

**Table 1:** Data extraction table

Metadata				Cardiovascular Disease Risk Assessment		
Author(s), year, country	Aim	Design	Sample size and target population	CVD risk assessment (delivery) a) Strategy b) Conducted by c) Location	CVD risk assessment (instrumentation) a) Measure(s) b) Adjusted for RA c) Frequency of use	Findings
Akenroye et al. (2017), USA [25]	Improve CVD screening rates by introducing an electronic reminder and decision support tool to the patient record	Quasi-experimental pre-post design	138 patients with RA pre intervention, 112 patients with RA post intervention	**a)** Electronic medical record reminder and decision support tool developed by a rheumatology consultant, a cardiology consultant, and a GP. Information sessions for rheumatology consultants regarding BP goals and definitions of abnormal lipid profiles **b)** Rheumatology consultants and GPs **c)** Hospital and Primary Care	**a)** FRS **b)** Not reported **c)** Not reported	Implementation of an electronic reminder did not improve CVD risk assessment CVD assessments were not performed in 93% (*n* = 104) of patients in the post intervention group
Ambrose et al. (2009), Ireland [26]	Assess the benefit of a shared care booklet for CVD risk screening and assessment	Pre-post intervention audit	80 patients with RA	**a)** Dual doctor and patient education and shared care intervention **b)** Rheumatology consultants **c)** Hospital	**a)** FRS **b)** Not reported **c)** Not reported	Pre intervention 80% (*n* = 64) of patients were inadequately assessed for CVD risk Improvement in measurement rates of CVD risk factors from 60–85% for BP, from 58–75% for lipid profiles, and from 55–80% for weight assessment after implementation of intervention
Bell and Rowe (2011), UK [27]	Determine the extent RA is identified as a risk factor for CVD in primary care and identify current risk assessment strategies	Survey	207 GPs	**a)** Not reported **b)** GPs **c)** Primary Care	**a)** Q RISK 2 **b)** Q RISK 2- RA included in algorithm **c)** Not reported	68% (*n* = 140) of GPs did not identify RA as a risk factor 85% (*n* = 175) of GPs did not perform routine CVD risk assessments in practice There was a significantly higher incidence of risk assessment rates (p value < 0.0001) among GPs who identified elevated CVD risk in RA or who had received education about RA related CVD risk
Emanuel et al. (2016), UK [28]	Compare differences in CVD risk factor assessment and management in patients with RA and inflammatory bowel disease in primary care	Prospective observational study	Electronic health records of 1,121 patients with RA	**a)** Not reported **b)** GPs **c)** Primary Care	**a)** FRS JBS Q RISK 2 **b)** FRS- not reported JBS- RA included in algorithm Q RISK 2- RA included in algorithm **c)** Not reported	Only 2% (*n* = 22) of patients with RA had a CVD risk assessment within 1 year of diagnosis Only 11% (*n* = 123) of patients with RA had a CVD risk assessment within 5 years of diagnosis
Gossec et al. (2013), France [29]	Assess the feasibility and the usefulness of a standardised CVD risk assessment form for use in the rheumatology outpatient department in patients with RA	Prospective observational study	110 patients with RA 22 rheumatology consultants	**a)** Standardised CVD risk assessment form including CVD risk assessment tool **b)** Rheumatology consultants **c)** Hospital and private practice	**a)** FRS **b)** Authors requested FRS results be × 1.5 **c)** Not reported	89% (*n* = 19) of consultants stated participation in this study improved their assessment of CVD risk in RA
Ikdahl et al. (2015), Norway [30]	Assess the implementation of European recommendations for annual CVD risk assessment in patients with RA in outpatient rheumatology clinics	Audit	Medical records of 1,142 patients with RA (612 from the regular rheumatology outpatient clinic and 530 from the arthritis clinic)	**a)** Rheumatology outpatient clinic- patient self-reporting on traditional risk factors on computer screens as part of CVD risk assessment Regular rheumatology outpatient clinic- not reported Arthritis clinic- structured MDT approach to CVD risk factor screening **b)** Regular rheumatology outpatient clinic- not reported Arthritis Clinic- rheumatology doctors, secretaries, and nurses **c)** Hospital	**a)** SCORE **b)** Not reported **c)** Annually	26.9% (*n* = 307) of patients in the rheumatology outpatient clinic had a CVD risk assessment 5.1% (*n* = 31) of patients in the regular rheumatology outpatient clinic had a CVD risk factor assessment versus 52.1% (*n* = 276) of patients in the arthritis clinic, [OR = 20.97 (95% CI = 14.0; 31.3)] P value < 0.001
Ikdahl et al. (2018), Norway [31]	Report the feasibility of a new CVD risk assessment programme as part of a nationwide quality assurance project, NOCAR	Observational Implementation study	4,483 patients with RA	**a)** Electronic data collection and display system, GoTreatIt Rheuma. HCP training on health promotion advice **b)** Computer data uploaded by secretaries and nurses, interpreted by rheumatology consultant **c)** Hospital	**a)** SCORE **b)** High CVD risk country chart used **c)** Annually	44.7% (*n* = 2,004) of eligible patients received a CVD risk assessment during the project period
Ladak et al. (2018), Canada [32]	Determine the frequency of CVD risk factor screening and treatment, and identify barriers to appropriate CVD risk management	Retrospective cohort study and survey	300 medical records from patients with RA 97 rheumatology consultants	**a)** Not reported **b)** Rheumatology consultants **c)** Hospital	**a)** FRS **b)** Not reported **c)** Not reported	63.9% (*n* = 61) of rheumatology consultants reported not adequately managing CVD risk despite 100% (*n* = 97) acknowledging it as a priority
Margo et al. (2014), Malta [33]	Determine if CVD risk assessment and management practices are in concordance with European recommendations	Audit	Medical records of 91 patients with RA	**a)** Not reported **b)** Not reported **c)** Hospital	**a)** SCORE **b)** SCORE result × 1.5 **c)** Annually	Only 31.9% (*n* = 29) of patients had data sufficient to undertake a CVD risk assessment
Raadsen et al. (2023), The Netherlands [34]	Assess CVD risk in patients with newly diagnosed RA and evaluate preventative treatment in high-risk patients	Cross-sectional observational study	125 patients with RA	**a)** Structured scheduling for CVD risk assessment when disease activity is low, direct communication to patient and GP **b)** Nurse **c)** Hospital	**a)** SCORE **b)** SCORE result × 1.5 **c)** Not reported	100% (*n* = 125) CVD risk assessment rate Preventative treatments remain insufficient in high-risk patients despite structured CVD risk assessments
Semb et al. (2016), Norway [35]	Examine to what degree two CVD risk calculators classify patients with inflammatory joint disease who have carotid plaque, and its effect of risk stratification	Cross-sectional observational study	201 patients with RA	**a)** Preventative combined Cardiology -Rheumatology Clinic, using carotid ultrasound **b)** Not reported **c)** Hospital	**a)** SCORE ACC/AHA **b)** SCORE result × 1.5 ACC/AHA- not reported **c)** Not reported	100% (*n* = 201) CVD risk assessment rate Application of the 1.5 multiplicator to the SCORE calculation only reclassified 3% (*n* = 6) of patients into the correct risk category after taking carotid atherosclerosis into consideration (not clinically significant)
Van den Oever et al. (2017), The Netherlands [36]	Assess the 10-year cardiovascular risk score and to identify treatment and undertreatment of cardiovascular risk factors	Prospective cross-sectional study	720 patients with RA	**a)** Implementation of cardiovascular risk assessment and management in RA, I-CaRe project **b)** Rheumatology doctors and research nurses **c)** Hospital and a rheumatology and rehabilitation centre	**a)** SCORE **b)** Add 15 years to patients’ age **c)** Annually	100% (*n* = 720) CVD risk assessment rate

*CVD* cardiovascular disease, *RA* rheumatoid arthritis, *GP* general practitioner, *BP* blood pressure, *FRS* framingham risk score, *Q RISK 2* the Q-RESEARCH cardiovascular risk algorithm version 2, *JBS* joint british societies CVD risk prediction score, *MDT* multidisciplinary team, *SCORE* systemic coronary risk evaluation, *OR* odds ratio, *CI* confidence interval, *ACC/AHA* American College of Cardiology/ American Heart Association

### Characteristics of sources of evidence

Of the 12 studies included, three originated from Norway [30, 31, 35], two from the UK [27, 28], two from the Netherlands [34, 36] and one from: Ireland [26], France [29], Malta [33], Canada [32], and the USA [25]. Studies were published between 2009 [26] and 2023 [34]. The designs of included studies were quasi-experimental [25], pre-post intervention audit [26], survey [27], prospective observational [28, 29], service audits [30, 33], observational implementational [31], retrospective cohort and survey [32], cross-sectional observational [34, 35], and prospective cross-sectional [36]. Sample sizes ranged from 22 [29] to 4,483 [31] participants. The combined target populations of included studies were patients with RA (*n* = 8,420), rheumatology consultants (*n* = 119), and General Practitioners (GPs)/Family Physicians (*n* = 207).

#### Strategies

Strategies to support the delivery of CVD risk assessments in patients with RA in routine care were reported in eight studies [25, 26, 29–31, 34–36], three of which reported 100% CVD risk assessment rates in routine care (*n* = 125) [34], (*n* = 201) [35], (*n* = 720) [36]. Each strategy adopted various system-based interventions with two approaches to support delivery: (a) multidisciplinary team (MDT) collaboration [25, 30, 31, 35, 36] and (b) education [25, 26, 31].

System-based interventions involved the use of an electronic medical record reminder with a decision support tool [25], a shared care booklet [26], a standardised CVD risk assessment form [29], a purposively designed CVD risk assessment clinic [30], an electronic data collection and display system [31], structured scheduling of CVD risk assessment [34], a purposively designed CVD prevention clinic [35], and a guideline implementation project [36]. Electronic systems were used by two studies as strategies to support delivery of CVD risk assessments in practice. Akenroye et al.’s [25] electronic medical record reminder was deemed unsuccessful by the authors as CVD risk assessments were not performed in 93% (*n* = 104) of patients. Ikdahl et al. [31] also reported the use of an electronic support system to support delivery of CVD risk assessments and cite an assessment rate of 44.7% (*n* = 2,004) in practice which, in their opinion, was deemed successful. Ambrose et al. [26] used a shared care booklet to improve rates of CVD risk factor screening and assessment in practice and found, after re-auditing their service, improved rates of risk factor screening (necessary to conduct CVD risk assessments) from 60–85% for blood pressure, 58–75% for lipid profiles, and from 55–80% for weight assessment. Ikdahl et al. [30] reported the use of a purposively designed clinic for CVD risk factor measurement and assessment yielding a 52.1% (*n* = 276) assessment rate. Raadsen et al. [34] also reported the use of a similar system-based approach with scheduling of CVD risk assessment clinic visits and reported a 100% (*n* = 125) CVD risk assessment rate. Semb et al. [35] used additional vascular imaging as part of their CVD risk assessment strategy. In their observational study 42% (*n* = 85) were found to have improved CVD risk stratification as a direct result of identifying carotid plaque on ultrasound compared to using a composite measure alone. Van den Oever et al. [36] designed the I-CaRe project to implement Dutch cardiovascular risk management recommendations [37]. As a result, van den Oever et al. [36] report a 100% (*n* = 720) CVD risk assessment rate in practice.

Of the studies that reported successful system-based interventions [30, 31, 34–36], four adopted an MTD approach to support delivery [30, 31, 35, 36]. Ikdahl et al. [30] developed a structured MDT clinic with defined roles for rheumatology doctors, nurses, and secretaries to implement European recommendations at that time [38]. They reported an increase of CVD risk assessment rates from 5.1% (*n* = 31) to 52.1% (*n* = 276) when patients were seen in the structured MDT clinic [OR = 20.97 (95% CI = 14.0; 31.3)]. Ikdahl et al. [31] presented results after implementation of a nation-wide quality assurance project involving a purposively designed CVD risk assessment programme. They also assigned duties to members of the rheumatology MDT and attribute its success primarily to this MDT approach. Semb et al. [35] reported the improvement of CVD risk stratification in their RA patient cohort because of specialised vascular imaging made possible by their cross disciplinary cardiology- rheumatology clinic. Van den Oever et al. [36] reported research nurses and rheumatology doctors undertake CVD risk assessments on all patients with RA as part of their Cardiovascular Risk Management in Rheumatoid Arthritis (I-Ca-Re) strategy, citing a 100% (*n* = 720) CVD risk assessment rate in routine care. Akenroye et al. [25] reported rheumatology consultants and GPs conducted CVD risk assessments collaboratively in their patients with RA, however despite implementing their electronic reminder, their intervention was deemed unsuccessful as assessment rates were only 7% (*n* = 8) in practice.

Education as a component of system-based interventions to support CVD risk assessment delivery was seen in three studies. Akenroye et al. [25] described the need for information sessions for participating rheumatologists. HCP and patient education was used in Ambrose et al.’s [26] shared care intervention to support CVD risk assessment delivery. The type, structure or content of this education was not described by the authors; however, a service audit eight months later demonstrated an improvement in measurement rates of CVD risk factors. Ikdahl et al. [31] reported the use of a CVD module and brief intervention training on smoking cessation and dietary advice for HCPs as part of their nationwide CVD risk assessment project.

#### Type of HCP conducting CVD risk assessments in patients with RA and the setting in which CVD risk assessments are carried out

Rheumatology doctors undertook CVD risk assessments on their own in four studies [26, 29, 32, 36], GPs undertook assessments in primary care in two studies [27, 28], and one study reported nurse led CVD risk assessment in routine care [34]. Another study reported that rheumatology consultants and GPs conducted CVD risk assessments on shared patients independently [25], and two studies reported involving a combination of rheumatology consultants, nurses, and secretaries in CVD risk assessment delivery [30, 31]. Information on who conducted CVD risk assessment was not reported in two papers [33, 35]. CVD risk assessments took place in the hospital setting [25, 26, 29–36], in primary care [25, 27, 28], in private practice [29], and in a rehabilitation centre [36].

### CVD risk assessment (instrumentation)

#### Composite CVD risk assessment measures in use and frequency of application

A variety of general population CVD risk assessment measures were used: the Systemic Coronary Risk Evaluation (SCORE) calculator [30, 31, 33–36]; the Framingham Risk Score (FRS) [25, 26, 28, 29, 32]; the Q Research Cardiovascular Risk (Q Risk) calculator [27, 28]; the Joint British Societies (JBS) score [28]; and the American College of Cardiology/American Heart Association (ACC/AHA) calculator [35]. Two studies stated the use of more than one composite measure of CVD risk in routine care [28, 35]. Only four studies reported on the frequency of CVD risk reassessment, which was conducted on a yearly basis [30, 31, 33, 36].

Of the 12 studies, four acknowledged the increased risk of CVD due to systemic inflammation related to RA [30, 31, 33, 36] and three acknowledged the impact of both, RA disease-specific and RA treatment-specific factors that result in increased CVD risk [27, 29, 32]. Interpretation of CVD risk assessment in terms of the impact of RA was discussed in three studies [29, 34, 36], where it was acknowledged that higher RA disease activity results in an increased CVD risk and therefore timing of the CVD assessment, for patients with RA, should be completed when disease is quiescent.

#### Adjustment of CVD risk assessment measure for RA

Of the six studies that reported using the SCORE tool [30, 31, 33–36], five stated the results were adjusted for RA [30, 31, 33–35] with varying methods including application of the 1.5 coefficient [33–35], using the high-risk country chart [31], or adding 15 years to the patients age [36]. Four of the five studies that reported using the FRS did not state if it was adjusted for RA [25, 26, 28, 32]. Gossec et al. [29] who also reported using the FRS reminded rheumatology consultants to apply the 1.5 multiplication factor to the FRS score as part of their study requirements; however, they acknowledged they were unable to ascertain if the consultants complied. Authors that cited the JBS and the Q RISK 2 as being in use in routine care [27, 28] did not need to adjust results to account for RA as both calculators include RA as an independent variable. The Q RISK 2 was used by the GP samples in both Bell and Rowe’s [27] survey and Emanuel et al.’s [28] observational study. The JBS calculator cited by Emanual et al. [28] also includes RA as an independent variable. The ACC/AHA calculator reported in Semb et al.’s [35] study does not have RA as a variable in its algorithm nor does the study indicate the results were adjusted to account for RA related CVD risk.

## Discussion

Results from this review demonstrated that although there is evidence that some strategies are used to support CVD risk assessment in patients with RA, extensive evidence establishing how HCPs conduct CVD risk assessments in practice is lacking. It was anticipated that a scoping review of the literature would yield the breadth of evidence necessary to map the strategies used in routine care. Many of the included studies focused on interventions to enhance current practices or reported CVD risk assessment as a by-product of an intervention to implement guidelines. Evidence such as retrospective chart reviews and standard operating protocols or procedures from professional rheumatology organisations might have allowed for more of a robust review of the current practice landscape.

From the studies included in this review, there is evidence to suggest that some strategies are used to support the delivery of CVD risk assessments in patients with RA [25, 26, 29–31, 34–36] with associated improved [29–31] or optimum [34–36] rates of assessment. Other evidence has been published that supports system-based programmes in the identification and measurement of CVD risk factors in patients with RA [39, 40], and system-based clinical decision supports for CVD risk assessment in the general population [41–43]. Tong et al. [44] suggests tools to assist HCPs in engaging in the CVD risk assessment conversation should also be developed with follow-up systems to facilitate CVD risk assessment and management. Of the 12 studies included in this review, four did not reference using any supportive strategy to deliver risk assessments in practice [27, 28, 32, 33]. Each of these four studies reported either low assessment rate levels [27, 28], inadequate management of CVD risk in general by the HCP [32], or low levels of CVD risk factor measurement [33]. Low CVD risk assessment rates in patients with RA results in missed opportunities for identification of CVD risk factors [45] and inhibits communication of individualised risk to the patient [46–48].

According to Gosh-Swaby et al. [49] patients who have the highest risk of developing CVD report the lowest awareness. Therefore, maximising assessment rates in practice is vital to ensure patients are aware of their individual risk, so treatments can be tailored accordingly. The supportive strategies identified in this review, albeit limited in numbers, reported improved or maximised rates of CVD assessment in routine care. By maximising rates of CVD risk assessment through supportive strategies and appropriate communication of CVD risk, more patients can become aware of their individual risk factors and can work with HCPs in initiating preventative therapy [49, 50].

Four of the five studies that used a system-based approach with MDT collaboration to support delivery of CVD risk assessments reported rates of between 44.7% -100% in practice [30, 31, 35, 36]. Despite adopting a cross disciplinary collaboration between rheumatologists and GPs to deliver CVD risk assessments in patients with RA, Akenroye et al. [25] reported assessment rates remained suboptimal at only 7% (*n* = 8). They suggest a possible reason for this was due to rheumatologists’ low awareness of RA related CVD risk. In contrast the broader literature has demonstrated that rheumatology consultants are aware of this risk but feel it’s the responsibility of the GPs to undertake CVD risk assessment in primary care [39, 46], demonstrating a lack of physician ownership in practice [51–53]. Three of these four studies involved nursing in their MDT collaboration [30, 31, 36]. One study cited independent nurse-led CVD risk assessment as part of routine care [34], without MTD collaboration but reported 100% CVD risk assessment rates. Studies exploring the impact of nurse-led care in patients with other chronic conditions have demonstrated improved outcomes compared to usual care [54–57]. Evidence has also been published to suggest nurses adopt a holistic and tailored approach to CVD risk assessment across a number of chronic conditions [58] with nurse-led CVD risk assessment programmes proving successful in the general population [59] primary care [60] and are well established in other chronic disease models of care [61–63]. Effective nurse-led CVD risk factor management programmes for patients with inflammatory disease have also been reported in the literature [64, 65]. According to European recommendations for the role of the nurse in inflammatory arthritis [66] rheumatology nurses should participate in comprehensive disease management and undertake extended roles to improve patient outcomes. Therefore, implementing nurse-led CVD risk assessment for patients with RA may prove effective in increasing rates and standardising delivery.

Strategies that used system-based interventions with an educational approach to support delivery of CVD risk assessments involved information sessions on CVD risk factor definitions and treatment goals [25], dual doctor and patient education interventions which were identified but not described in the text [26] and educating HCPs on health promotion advice [31]. Only one of these studies reported having satisfactory CVD risk assessment rates in practice of 44.7% (*n* = 2,004) [31]. Education highlighting the awareness of RA as an independent CVD risk factor was not seen in any of the included studies. Perhaps because the included studies were undertaken largely in hospital settings with rheumatology consultants who, according to Nguyen-Oghalai et al. [46], are aware of RA related CVD risk, therefore education of HCPs as a component of a CVD risk assessment framework might not be deemed necessary. Bell and Rowe’s [27] survey highlighted the importance of GP education where it was noted that GPs who had received education about RA, or who identified RA as a risk factor were significantly more likely to undertake a CVD risk assessment on patients with RA (*p* < 0.0001). Patient education was only seen in combination with HCP education in one study [26]. Protocols defining integrated roles for both rheumatology teams and GPs when CVD risk assessing patients with RA is essential but should also include patient education to compliment clinical discussions [67]. Only two studies reported incorporating both MDT collaboration and HCP education [25, 31] with varying results.

A number of CVD risk assessment measures were reported in use. Six of the European studies [30, 31, 33–36] reported using the SCORE calculator which was initially validated for use across 12 European cohorts [68]. Both the USA and Canadian studies [25, 32] cited the use of the FRS, mirroring the American validation cohort [69]. Both UK based studies [27, 28] reported using the Q RISK measure, aligning with the initial British derivation and validation cohort [70]. It appears, from this review, HCPs favour CVD risk assessment measures developed in cohorts similar to their patient populations. Measurements developed for and validated in specific countries may not be suitable for use in other countries unless adequate testing and validation has occurred, as differences in the prevalence of traditional risk factors across countries can affect risk scores, resulting in inaccurate risk predictions [71].

The most frequently cited assessment measure was the SCORE calculator [30, 31, 33–36] with five studies reporting score adjustment to account for RA [31, 33–36]. The FRS was also cited in use [25, 26, 28, 29, 32] but without reference to adjustment for RA. The FRS algorithm significantly underestimates CVD risk in RA patients, particularly older patients with positive serology and patients with persistently elevated inflammatory markers [72, 73]. Therefore, it is essential when using a general population calculator such as the FRS to adjust scores to account for RA related CVD risk [16].

This review found no RA disease specific CVD risk assessment measures in use in routine care. Disease specific calculators have been developed that include traditional CVD risk factors in their algorithm with the addition of disease specific variables such as clinical activity, corticosteroid use, and elements of functional ability (see in Online Resource 1) which have notable effects on CVD risk [74]. However, despite this, RA specific CVD risk calculators are not superior to general population CVD risk calculators in estimating future CVD risk in patients with RA [75, 76] and as a result are not recommended for use in patients with RA over modified general population risk calculators [16].

Of the five composite measures mentioned in the included studies, two include RA disease as an independent variable (Q RISK and JBS), [27, 28], recognizing the impact of inflammatory disease on CVD risk. None of the measures incorporated C-Reactive Protein, an inflammatory mediator, which rises in states of inflammation including flaring RA disease [77, 78]. Only one measure (Q RISK) captured corticosteroid use, recognizing the impact of medication on CVD risk. Of note, composite measures included in our review did not make a distinction between the role of inflammation from RA disease and the role of RA treatment (e.g., corticosteroids) in increased CVD risk.

## Strengths and limitations

The search strategy for this review was not limited to a specific period to help retrieval and avoid reporting bias. The search field was broadened by using a number of platforms to search for sources of evidence including electronic databases, trial registries, and the grey literature. The double screening process helped ensure that relevant studies were not missed. As for limitations it is possible that studies reporting CVD risk screening practices might have also conducted CVD risk assessments as part of routine care but did not report it as a separate/discrete element.

## Conclusions

Findings of this scoping review identified a variety of system-based interventions to support the delivery of CVD risk assessments in patients with RA, operationalised in different ways using one, or a combination of, two approaches: (a) MDT collaboration, and (b) education. Various HCPs deliver CVD risk assessments in different settings including the hospital, private practice, rehabilitation units and primary care. A number of general population CVD risk assessment measurements were cited in use by studies in this review, with and without adjustment for RA. This review demonstrates that although several strategies to support the delivery of CVD risk assessments in patients with RA are cited in use in the literature, there is limited evidence to suggest a standardised model has been applied in practice. This review has identified a gap in the literature of robust evidence detailing the CVD risk assessment practices of HCPs in the routine care of patients with RA. Research needs to be undertaken to establish the extent to which HCPs are CVD risk assessing their patients with RA as part of routine care.

### Supplementary Information

Below is the link to the electronic supplementary material.

Supplementary file1 (DOCX 23 KB)

Supplementary file2 (DOCX 26 KB)

Supplementary file3 (DOCX 15 KB)

Supplementary file4 (DOCX 21 KB)

## Data Availability

The author confirms that all data generated or analysed during this study are available in supplementary information files 1–4.
