# Supplementary material for: Cardiovascular disease risk assessment in patients with rheumatoid arthritis: A scoping review
Source: Clin Rheumatol. 2024 May 11;43(7):2187–202. doi: 10.1007/s10067-024-06996-3 (PMC11189331; doi:10.1007/s10067-024-06996-3)
Supplement: Supplementary file 1 — Supplementary file1 (DOCX 23 KB) [file 10067_2024_6996_MOESM1_ESM.docx]

**Online Resource 1: CVD risk prediction measures used in patients with RA and algorithm variables.**

| Instrument, Author, year | Algorithm variables | | | | | | | | | | | | | | | | | | | | |
| --- | --- | --- | --- | --- | --- | --- | --- | --- | --- | --- | --- | --- | --- | --- | --- | --- | --- | --- | --- | --- | --- |
|  | Age | Fm Hx | Gen | A fib | SBP/ HTN | CRP | Smoking | No of Cigs | Lipoproteins | BMI | SD | DM | Eth | CKD | RA | GC | SLE | Mig | MI | A-P Rx | ED |
| RRS  Ridker et al. (2007) | 🗸 | 🗸 | 🗸 | X | SBP | 🗸 | 🗸 | X | TC/HDL | X | X | X | X | X | X | X | X | X | X | X | X |
| PROCAM  Assmann et al. (2002) | 🗸 | 🗸 | X | X | SBP | X | 🗸 | X | HDL/LDL/Trig | X | X | 🗸 | X | X | X | X | X | X | X | X | X |
| FRS  D’Agostino et al. (2008) | 🗸 | X | 🗸 | X | HTN/SBP | X | 🗸 | X | TC/HDL | X | X | 🗸 | X | X | X | X | X | X | X | X | X |
| SCORE*  Conroy et al. (2003) | 🗸 | X | 🗸 | X | SBP | X | 🗸 | X | TC | X | X | X | X | X | X | X | X | X | X | X | X |
| ACC/AHA  Goff et al. (2014) | 🗸 | X | 🗸 | X | HTN/SBP | X | 🗸 | X | TC/HDL | X | X | 🗸 | 🗸 | X | X | X | X | X | X | X | X |
| JBS3 score**  JBS3 Board (2013) | 🗸 | 🗸 | 🗸 | 🗸 | HTN/SBP | X | 🗸 | 🗸 | TC/HDL | 🗸 | 🗸 | 🗸 | 🗸 | 🗸 | 🗸 | X | X | X | X | X | X |
| Q RISK 3**  Hippisley-Cox et al. (2017) | 🗸 | 🗸 | 🗸 | 🗸 | HTN/SBP | X | 🗸 | 🗸 | TC/HDL | 🗸 | 🗸 | 🗸 | 🗸 | 🗸 | 🗸 | 🗸 | 🗸 | 🗸 | 🗸 | 🗸 | 🗸 |
| ERS- RA  Solomon et al. (2015) | 🗸 | X | 🗸 | X | HTN/SBP | X | 🗸 | X | hyperlipidaemia | X | X | 🗸 | X | X | RA duration  CDAI  HAQ | 🗸 | X | X | X | X | X |
| ATTACC-RA  Crowson et al. (2017) | 🗸 | X | 🗸 | X | HTN | X | 🗸 | X | TC/HDL | X | X | X | X | X | RA duration  Serology  DAS28 (ESR)  HAQ | X | X | X | X | X | X |

Fm Hx: Family History, Gen: Gender, A Fib: Atrial Fibrillation, SBP: Systolic Blood Pressure, HTN: Hypertension, Cigs: Cigarettes, BMI: Body Mass Index, SD: Social Deprivation, DM: Diabetes Mellitus, Eth: Ethnicity, CKD: Chronic Kidney Disease, RA: Rheumatoid Arthritis, GC: Glucocorticoid**,** SLE: Systemic Lupus Erythematosus, Mig: Migraine, MI: Mental Illness, A-P Rx: Anti-Psychotic Medication, ED: Erectile Dysfunction, RRS: Reynolds Risk Score, TC: Total Cholesterol, HDL: High Density Lipoprotein, PROCAM: The Munster Heart Study calculator formerly known as the Prospective Cardiovascular Munster Study, LDL: Low Density Lipoprotein, Trig: triglycerides, FRS: Framingham Risk Score, SCORE: The Systemic Coronary Risk Evaluation, ACC/AHA: The American College of Cardiology and the American Heart Association, JBS3: Joint British Societies Score (version 3), Q RISK 3: The Q-RESEARCH Cardiovascular Risk Score (version 3), ERS-RA: Expanded Risk Score for Rheumatoid Arthritis, CDAI: Clinical Disease Activity Index, HAQ: Health Assessment Questionnaire, ATACC-RA: A Transatlantic Cardiovascular risk Calculator for Rheumatoid Arthritis, DAS28: Disease Activity Score 28 Joint Count, ESR: Erythrocyte Sedimentation Rate.

*Both high and low risk county charts.

** Risk calculator algorithm updated over time with newer versions including additional variables. The most recently published calculator was chosen for inclusion into the above table to demonstrate algorithm variables.

References

1. Ridker PM, Buring JE, Rifai N, Cook NR (2007) Development and validation of improved algorithms for the assessment of global cardiovascular risk in women: the Reynolds risk score. Jama 297(6):611–9. [https://doi.org/1 0.1001/jama.297.6.611](https://doi.org/1%200.1001/jama.297.6.611)
2. Assmann G, Schulte H, Cullen P, Seedorf U (2007) Assessing risk of myocardial infarction and stroke: new data from the Prospective Cardiovascular Münster (PROCAM) study. Eur J Clin Invest 37(12):925-32. https://doi:10.1111/j.1365-2362.2007.01888.x PMID: 18036028
3. D'Agostino RB Sr, Vasan RS, Pencina MJ, Wolf PA, Cobain M, Massaro JM, Kannel WB (2008) General cardiovascular risk profile for use in primary care: the Framingham Heart Study. Circ 117(6), 743-753. https://doi:10.1161/CIRCULATIONAHA.107.699579 PMID: 18212285
4. Conroy RM, Pyörälä K, Fitzgerald AP, Sans S, Menotti A, De Backer G, De Bacquer D, Ducimetière P, Jousilahti P, Keil U, Njølstad I, Oganov RG, Thomsen T, Tunstall-Pedoe H, Tverdal A, Wedel H, Whincup P, Wilhelmsen L, Graham IM; SCORE project group (2003) Estimation of ten-year risk of fatal cardiovascular disease in Europe: the SCORE project. Eur Heart J 24(11):987-1003. https://doi:10.1016/s0195-668x(03)00114-3 PMID: 12788299
5. Goff DC Jr, Lloyd-Jones DM, Bennett G, Coady S, D’Agostino RB Sr, Gibbons R, Greenland P, Lackland DT, Levy D, O’Donnell CJ, Robinson JG, Schwartz JS, Shero ST, Smith SC Jr, Sorlie P, Stone NJ, Wilson PWF (2014) ACC/AHA guideline on the assessment of cardiovascular risk: a report of the American College of Cardiology/American Heart Association Task Force on Practice Guidelines. Circ 129(suppl 2):S49-S73.
6. Joint British Societies’ consensus recommendations for the prevention of cardiovascular disease (JBS3) Joint British Societies Board (2014) Heart 100:ii1–ii67. https://doi:10.1136/heartjnl-2014-305693
7. Hippisley-Cox J, Coupland C, Brindle P (2017) Development and validation of QRISK3 risk prediction algorithms to estimate future risk of cardiovascular disease: prospective cohort study. Bri Med J 357:j2099 https://doi:10.1136/bmj.j2099
8. Solomon DH, Greenberg J, Curtis JR, Liu M, Farkouh ME, Tsao P, Kremer JM, Etzel CJ (2015) Derivation and internal validation of an expanded cardiovascular risk prediction score for rheumatoid arthritis: a Consortium of Rheumatology Researchers of North America Registry Study. Arthritis Rheumatol 67(8):1995-2003. https://doi:10.1002/art.39195 Erratum in: Arthritis Rheumatol. 2016 Feb;68(2):515. PMID: 25989470.
9. Crowson CS, Rollefstad S, Kitas GD, van Riel PL, Gabriel SE, Semb AG; A Trans-Atlantic Cardiovascular Risk Consortium for Rheumatoid Arthritis (ATACC-RA) (2017) Challenges of developing a cardiovascular risk calculator for patients with rheumatoid arthritis. PLoS One 12(3):e0174656. https://doi:10.1371/journal.pone.0174656 Erratum in: PLoS One. 2017 Apr 7;12 (4):e0175605. PMID: 28334012; PMCID: PMC5363942.
