# Supplementary material for: Cardiovascular disease risk assessment in patients with rheumatoid arthritis: A scoping review
Source: Clin Rheumatol. 2024 May 11;43(7):2187–202. doi: 10.1007/s10067-024-06996-3 (PMC11189331; doi:10.1007/s10067-024-06996-3)
Supplement: Supplementary file 3 — Supplementary file3 (DOCX 15 KB) [file 10067_2024_6996_MOESM3_ESM.docx]

**Online Resource 3: Relevant keywords and Subject Headings**

| Key Words | Cardiovascular Disease/ Heart Attack/ Stroke/ Peripheral Vascular Disease | Risk Assessment | Rheumatoid Arthritis |
| --- | --- | --- | --- |
| CINAHL subject headings | Cardiovascular Diseases  Heart Diseases  Coronary Disease  Myocardial Ischemia  Myocardial Infarction  Angina, Stable  Angina, Unstable  Angina, Pectoris  Coronary Stenosis  Carotid Stenosis  Stroke  Ischemic Stroke  Cerebrovascular Disorders  Cerebral Ischemia, Transient  Peripheral Vascular Diseases  Heart Disease Risk Factors  Cardiometabolic Risk Factors  Cardiovascular Risk Factors | Risk Assessment  Risk Management  Patient Assessment  Clinical Assessment Tools  Predictive Validity | Arthritis, Rheumatoid  Arthritis |
| Medline  MeSH terms | Cardiovascular Diseases  Heart Diseases  Coronary Disease  Myocardial Ischemia  Myocardial Infarction  Angina, Stable  Angina, Unstable  Angina, Pectoris  Coronary Stenosis  Carotid Stenosis  Stroke  Ischemic Stroke  Cerebrovascular Disorders  Ischemic Attack, Transient  Peripheral Vascular Diseases  Peripheral Arterial Disease  Heart Disease Risk Factors  Cardiometabolic Risk Factors | Risk Assessment  Risk Management  Risk | Arthritis, Rheumatoid  Rheumatic Diseases  Arthritis |
