# Supplementary material for: Cardiovascular disease risk assessment in patients with rheumatoid arthritis: A scoping review
Source: Clin Rheumatol. 2024 May 11;43(7):2187–202. doi: 10.1007/s10067-024-06996-3 (PMC11189331; doi:10.1007/s10067-024-06996-3)
Supplement: Supplementary file 4 — Supplementary file4 (DOCX 21 KB) [file 10067_2024_6996_MOESM4_ESM.docx]

**Online Resource 4: Search Strategies**

**CINAHL search strategy**

Interface

 - EBSCOhost Research Databases

Search Screen

 - Advanced Search

Database

 - CINAHL Plus with Full Text

Limiters: Language: English

Expanders - Apply equivalent subjects

Search modes - Boolean/Phrase

S1: TI OR AB ( “Cardi* Disease*” OR CVD OR “Heart Disease*” OR “Coronary Disease*” OR CHD OR “Myocardial Ischemi*” OR “Myocardial Infarct*” OR MI OR “Coronary Stenosis” OR “Carotid Stenosis” OR CVA OR “Cerebr* Disorder*” OR TIA OR “Peripheral Vascular Disease*” OR PVD OR (MH "Heart Diseases") OR (MH "Myocardial Ischemia") OR (MH "Coronary Disease") OR (MH "Myocardial Infarction") OR (MH "Angina, Stable") OR (MH "Angina, Unstable") OR (MH "Angina Pectoris") OR (MH "Coronary Stenosis") OR (MH "Carotid Stenosis") OR (MH "Cerebral Ischemia, Transient") OR (MH "Cerebrovascular Disorders") OR (MH "Stroke") OR (MH "Ischemic Stroke") OR (MH "Peripheral Vascular Diseases") OR (MH "Cardiovascular Diseases") OR (MH "Heart Disease Risk Factors") OR (MH Cardiometabolic Risk Factors") OR (MH "Cardiovascular Risk Factors") )

S2: TI OR AB ( (MH “Risk Assessment”) OR (Risk N3 Assess*) OR (MH “Risk Management”) OR (Risk N3 Manag*) OR (MH “Patient Assessment”) OR “Patient Assess*” OR (MH “Clinical Assessment tools”) OR “Assess* Algorithm*” OR Predict* OR (MH “Predictive Validity”) )

S3: TI OR AB ( (MH “Arthritis, Rheumatoid”) OR ("Rheumatoid Arthritis") OR (MH “Arthritis”) OR Arthritis )

S4: S1 AND S2 AND S3

**MEDLINE search strategy**

Interface

- EBSCOhost Research Databases

Search Screen

- Advanced Search

Database

- MEDLINE

Limiters - Language: English

Expanders - Apply equivalent subjects

Search modes - Boolean/Phrase

S1: TI OR AB ( “Cardi* Disease*” OR CVD OR “Heart Disease*” OR “Coronary Disease*” OR CHD OR “Myocardial Ischemi*” OR “Myocardial Infarct*” OR MI OR “Coronary Stenosis” OR “Carotid Stenosis” OR CVA OR “Cerebr* Disorder*” OR TIA OR “Peripheral Vascular Disease*” OR PVD (MH "Cardiovascular Diseases") OR (MH "Heart Diseases") OR (MH "Coronary Disease") OR (MH "Myocardial Ischemia") OR (MH "Myocardial Infarction") OR (MH "Angina Pectoris") OR (MH "Angina, Stable") OR (MH "Angina, Unstable") OR (MH "Coronary Stenosis") OR (MH "Carotid Stenosis") OR (MH "Stroke") OR (MH "Cerebrovascular Disorders") OR (MH "Ischemic Stroke") OR (MH "Ischemic Attack, Transient") OR (MH "Peripheral Vascular Diseases") OR (MH "Peripheral Arterial Disease") OR (MH "Heart Disease Risk Factors") OR (MH "Cardiometabolic Risk Factors") )

S2: TI OR AB (MH "Risk Assessment") OR (MH "Risk Management") OR (Risk N3 Assess*) OR (Risk N3 Manag*) OR “Patient Assess*” OR “Assess* Algorithm*” OR Predict*

S3: TI OR AB (MH "Arthritis, Rheumatoid") OR (MH "Rheumatic Diseases") OR (MH "Arthritis") OR ("Rheumatoid Arthritis") OR Arthritis

S4: S1 AND S2 AND S3

**Scopus search strategy**

Interface

- Elsevier

Search Screen

- Document Search

Database

- Scopus

Limiters: English Language

#1: TITLE-ABS “Cardi* Disease*” OR CVD OR “Heart Disease*” OR “Coronary Disease*” OR CHD OR “Myocardial Infarct*” OR “Coronary Stenosis” OR “Carotid Stenosis” OR CVA OR “Cerebr* Disorder*” OR TIA OR “Peripheral Vascular Disease*” OR PVD

#2: TITLE-ABS (Risk W/3 Assess*) OR “Risk Management” OR (Risk W/3 Manag*) OR “Patient Assess*” OR “Clinical Assessment tools” OR “Assess* Algorithm*” OR Predict*

#3 TITLE-ABS "Rheumatoid Arthritis" OR Arthritis

#1 AND #2 AND #3

**Cochrane Database of Systematic Reviews search strategy**

Interface

 -  Wiley InterScience Website

Search Screen

 - Advanced Search

Database

 - Cochrane Library

Expanders - Explode all trees for MeSH descriptors, word variations.

Search modes - Boolean/Phrase

#1: TI OR AB OR KW with word variations Cardi* NEXT Disease* OR CVD OR Heart NEXT Disease* OR Coronary NEXT Disease* OR CHD OR Myocardial NEXT Ischemi* OR Myocardial NEXT Infarct* OR MI OR Coronary NEXT Stenosis OR Carotid NEXT Stenosis OR CVA OR Cerebr* NEXT Disorder* OR TIA OR Peripheral NEXT Vascular NEXT Disease* OR PVD

#2: MeSH descriptor: [Cardiovascular Diseases] explode all trees

#3: MeSH descriptor: [Cerebrovascular Disorders] explode all trees

#4: MeSH descriptor: [Peripheral Vascular Diseases] explode all trees

#5: #1 OR #2 OR #3 OR #4

#6: TI OR AB OR KW with word variations (Risk N3 Assess*) OR (Risk N3 Manag*) OR Patient NEXT Assess* OR Assess* NEXT Algorithm* OR Predict*

#7: MeSH descriptor: [Risk Assessment] explode all trees

#8: MeSH descriptor: [Risk Management] explode all trees

#9: #6 OR #7 OR #8

#10: TI OR AB OR KW with word variations Rheumatoid NEXT Arthritis OR Arthritis

#11: MeSH descriptor: [Arthritis, Rheumatoid] explode all trees

#12: MeSH descriptor: [Rheumatic Diseases] explode all trees

#13: MeSH descriptor: [Arthritis] explode all trees

#14: #10 OR #11 OR #13

#15: #5 AND #9 AND #14

**Web of Science search strategy**

Interface

- Clarivate

Search Screen

- Document Search

Database

- All Databases, all collections

Limits- English Language

Search Modes- Boolean/ Phrase

#1: TI OR AB =(“Cardi* Disease*” OR CVD OR “Heart Disease*” OR “Coronary Disease*” OR CHD OR “Myocardial Ischemi*” OR “Myocardial Infarct*” OR MI OR “Coronary Stenosis” OR “Carotid Stenosis” OR CVA OR “Cerebr* Disorder*” OR TIA OR “Peripheral Vascular Disease*” OR PVD)

#2: TI OR AB = ((Risk NEAR/3 Assess*) OR (Risk NEAR/3 Manag*) OR “Patient Assess*” OR “Assess* Algorithm*” OR Predict* ))

#3: TI OR AB = ("Rheumatoid Arthritis" OR Arthritis)

#1 AND #2 AND #3

**Academic Search Complete search strategy**

Interface

 - EBSCOhost Research Databases

Search Screen

 - Advanced Search

Database

- Academic Search Complete

Limiters: Language: English

Expanders - Apply equivalent subjects

Search modes - Boolean/Phrase

S1: TI OR AB ( “Cardi* Disease*” OR CVD OR “Heart Disease*” OR “Coronary Disease*” OR CHD OR “Myocardial Ischemi*” OR “Myocardial Infarct*” OR MI OR “Coronary Stenosis” OR “Carotid Stenosis” OR CVA OR “Cerebr* Disorder*” OR TIA OR “Peripheral Vascular Disease*” OR PVD OR (MH "Heart Diseases") OR (MH "Myocardial Ischemia") OR (MH "Coronary Disease") OR (MH "Myocardial Infarction") OR (MH "Angina, Stable") OR (MH "Angina, Unstable") OR (MH "Angina Pectoris") OR (MH "Coronary Stenosis") OR (MH "Carotid Stenosis") OR (MH "Cerebral Ischemia, Transient") OR (MH "Cerebrovascular Disorders") OR (MH "Stroke") OR (MH "Ischemic Stroke") OR (MH "Peripheral Vascular Diseases") OR (MH "Cardiovascular Diseases") OR (MH "Heart Disease Risk Factors") OR (MH Cardiometabolic Risk Factors") OR (MH "Cardiovascular Risk Factors") )

S2: TI OR AB ( (MH “Risk Assessment”) OR (Risk N3 Assess*) OR (MH “Risk Management”) OR (Risk N3 Manag*) OR (MH “Patient Assessment”) OR “Patient Assess*” OR (MH “Clinical Assessment tools”) OR “Assess* Algorithm*” OR Predict* OR (MH “Predictive Validity”) )

S3: TI OR AB ( (MH “Arthritis, Rheumatoid”) OR ("Rheumatoid Arthritis") OR (MH “Arthritis”) OR Arthritis )

S4: S1 AND S2 AND S3

**Grey literature database search strategies**

**Trip Pro**

Advanced search screen

Document search: cardiovascular, “rheumatoid arthritis”, “risk management” with any of these words: cerebrovascular, “peripheral vascular”, arthritis, “risk assessment”, “risk manage”, algorithm, predict

**OAIster**

Search Results Display: Hide duplicates

Expand search with related terms: Off

Held By Library: Libraries Worldwide

Databases: OAIster

Language: English

#1 kw (“Cardi* Disease*” OR CVD OR “Heart Disease*” OR “Coronary Disease*” OR CHD OR “Myocardial Ischemi*” OR “Myocardial Infarct*” OR MI OR “Coronary Stenosis” OR “Carotid Stenosis” OR CVA OR “Cerebr* Disorder*” OR TIA OR “Peripheral Vascular Disease*” OR PVD)

#2 kw ("Risk Assessment” OR Risk Assess* OR “Risk Management” OR “Patient Assessment” OR “Patient Assess*” OR “Clinical Assessment tools” OR “Assess* Algorithm*” OR Predict*)

#3 kw (“Rheumatoid Arthritis” OR arthritis)

#1 AND #2 AND #3 = 371

**Base**

Bielefeld University Library

Search Screen: Advanced Search

Document type: All

Terms of Re-use/Licences: All

Access: Open access, non-open access, unknown

Language: English
Verbatim search: On
Boosting: Open access documents

Title: Cardiovascular Disease

Title: Rheumatoid Arthritis

Title: Risk Assessment

**Major medical clearing house search strategies**

1: Lenus

Databases: All of Lenus, the Irish Health Repositry

Search Strategy: Rheumatoid Arthritis AND Cardiovascular Disease AND Risk Assessment

2: The National Guideline Clearinghouse

Search Page: Basic Search

Strategy: Rheumatoid Arthritis AND Cardiovascular Disease AND Risk Assessment

3: The Guidelines International Network

Search Strategy: Rheumatoid Arthritis AND Cardiovascular Disease AND Risk Assessment

**ILAR professional organisations search strategy**

**1: American College of Rheumatology (ACR)**

Section search- Practice & Quality- Clinical Support-

Clinical Practice Guidelines

Guidance Documents

Quality Measurement

Criteria

**2: African League of Associations for Rheumatology (AFLAR)**

Section search- Education and Research- Publications- African Journal of Rheumatology

**3: Asia Pacific League of Associations for Rheumatology (APLAR)**

Section search- Publication- Published Journal

Search Bar Search

Rheumatoid Arthritis

Cardiovascular Disease

**4: European Alliance of Associations for Rheumatology, formally known as the European League Against Rheumatism (EULAR)**

Section search- Educations- EULAR publications

Search Bar Search

Rheumatoid Arthritis

Cardiovascular Disease

Section search- Quality of Care

EULAR Recommendations

Published recommendations

Classification and Diagnosis Criteria/ response criteria

Recommendations for management

EULAR/ ACR Collaborative Projects

**5: Pan American League of Associations for Rheumatology** **(PANLAR)**

Section search- Resource Centre

Search Bar Search

Rheumatoid Arthritis

Cardiovascular Disease
